# Epidemiological profile and clinical characteristics of 491 Brazilian patients with neurofibromatosis type 1

**DOI:** 10.1002/brb3.2599

**Published:** 2022-05-04

**Authors:** Luiz Guilherme Darrigo Junior, Victor Evangelista de Faria Ferraz, Marina Candido Visontai Cormedi, Luissa Hikari Hayashi Araujo, Mariana Prado Silva Magalhães, Rafaella Curis Carneiro, Luis Henrique Nunes Sales, Mendel Suchmacher, Karin Soares Cunha, Aguinaldo Bonalumi Filho, David Rubem Azulay, Mauro Geller

**Affiliations:** ^1^ Departament of Pediatrics Ribeirão Preto Medical School University of São Paulo São Paulo São Paulo Brazil; ^2^ Departament of Genetics Ribeirão Preto Medical School University of São Paulo São Paulo São Paulo Brazil; ^3^ Immunology Department Fundação Educacional Serra dos Órgãos (UNIFESO), Rio de Janeiro Rio de Janeiro Brazil; ^4^ Department of Pathology and Post‐graduation Program in Pathology School of Medicine Fluminense Federal University, Rio de Janeiro Rio de Janeiro Brazil; ^5^ Hospital Naval Marcilio Dias (HNMD), Rio de Janeiro Rio de Janeiro Brazil; ^6^ Dermatology Service Prof. Rubem David Azulay Dermatology Institute, Rio de Janeiro Rio de Janeiro Brazil; ^7^ Clinical Immunology Instituto de Pós‐Graduação Médica Carlos Chagas (IPGMCC), Rio de Janeiro Rio de Janeiro Brazil

**Keywords:** gliomas, malignant peripheral nerve sheath tumor, neurofibroma, neurofibromatosis type 1, plexiform neurofibroma

## Abstract

**Background:**

Neurofibromatosis type 1 (NF1) is a chronic and progressive autosomal dominant genetic and sporadic disease characterized by cutaneous and neurological abnormalities. Plexiform neurofibroma (PN), a significant cause of clinical complications in NF‐1, is a benign tumor of the peripheral nerve sheath that involves multiple nerve fascicles. Although there is an important number of patients who are affected by NF1 in Brazil, there is little data on the behavior of the disease in the national literature as well as in other low‐ and middle‐income countries.

**Methods:**

We performed a retrospective analysis of 491 patients with NF1 followed at two reference centers in Brazil.

**Results:**

Approximately 38% of patients had PNs, resulting in reduced life quality. The median patient age with PNs was 30 years (range: 6 to 83 years). Head and neck, and extremity were the main affected locations with 35.8 and 30.6%, respectively. PNs were classified as asymptomatic in 25.1% of patients, while 52.5% presented symptomatic and inoperable tumors. The most common manifestations related to PNs were disfigurement and orthopedic involvement. Twenty patients developed neoplasms and ten (50%) presented with malignant peripheral nerve sheath tumors (MPNST). The prevalence of MPNST in our study was 2.9%.

**Conclusions:**

Patients with NF1 experience clinically significant morbidity, especially when it is associated with PN. Though there are many patients affected by NF1 in Brazil and other low‐ and middle‐income countries, there is little data available in the corresponding literature. Our results are comparable to the previous results reported from higher‐income countries and international registries.

## 1 INTRODUCTION

Neurofibromatosis type 1 (NF1) is a common autosomal dominant multisystem genetic and sporadic disease with complete penetrance and highly variable expression, resulting in a broad spectrum of clinical manifestations and is considered one of the most common genetic diseases with a predisposition to malignancy. (Darrigo Junior et al., 2008; Gutmann et al., 2017) The incidence of NF1 is approximately 1 in 2700 people, affecting all ethnicities and having an identical correlation between men and women (Evans et al., 2010).

It is caused by a germline mutations affecting the *NF1* gene, which encodes the protein neurofibromin, a GTPase‐activating protein (GAPs), which activates the intrinsic GTPase of *Ras* and negatively regulates its role in signal transduction (Bezniakow et al., 2014; Griffiths et al., 2007; Yap et al., 2014). Thus, loss of neurofibromin expression, as observed in different tumors associated with NF1, results in high levels of activated *Ras*, leading to increased cell growth and survival through hyperactivation of *Ras* (Gutmann et al., 2017). Neurofibromin is a 220 kDa cytoplasmic protein comprising a 300‐amino acid sequence with significant homology to domains found in GAPs and is predominantly expressed in neurons, Schwann cells, oligodendrocytes, and leukocytes (Declue et al., 1991; Gutmann et al., 2017; Xu et al., 1990).

Diagnostic criteria of NF1 were established at the National Institutes of Health (NIH) Consensus Development Conference in 1987 and were revised. The new diagnostic criteria for NF require the presence of two or more of the following criteria: (a) six or more cafe‐au‐lait macules (CALMs) >0.5 cm in prepubertal children or >1.5 cm in postpubertal individuals; (b) freckling; (c) two or more cutaneous neurofibromas or one plexiform neurofibroma (PN); (d) two or more Lisch nodules or two or more choroidal abnormalities; (e) one optic pathway glioma; (f) a distinctive osseous lesion such as: sphenoid dysplasia; anterolateral bowing of tibia (tibial dysplasia); or pseudarthrosis of a long bone; (g) a pathogenic NF1 gene variant and (h) a first‐degree relative with NF1 (Legius et al., 2021; Neurofibromatosis, 1988). The onset and severity of nearly all clinical features of NF1 are age‐dependent and highly variable, even within affected families (Debella et al., 2000). As observed, the clinical phenotypes associated with NF1 are numerous and include both tumor and nontumor symptoms. One of the striking clinical manifestations of NF1 is the development of PNs (Prada et al., 2012). PNs are congenital lesions that occur commonly in individuals with NF1, affecting approximately 30% of all patients. (Waggoner et al., 2000) They are defined as benign neoplasms that affect multiple fascicles of a nerve or a nervous plexus and present a significantly increased risk of developing malignant peripheral nerve sheath tumors (MPNSTs).

PNs grow during the early years of life. Although some of them remain stable, most of them show progressive growth. They cause significant deformity and morbidity owing to the impact on critical structures (Korf, 1999; Prada et al., 2012; Packer et al., 2002). Due to the morbidity and mortality associated with PN, these tumors are currently the subject of numerous studies (Dombi et al., 2016; Gross & Widemann, 2020; Packer et al., 2002). Thus, a better understanding of PN's clinical and epidemiological aspects, especially in low‐ and middle‐income countries, is necessary for appropriate health promotion for patients with NF1 outside higher‐income countries. The aim of this study was to identify through retrospective data the main clinical and epidemiological characteristics of PNs in patients affected by NF1 in Brazil.

## 2 MATERIALS AND METHODS

We conducted a multicenter observational study that involved the retrospective review of medical records of patients diagnosed with NF1 attended at two referral centers in Brazil: Ribeirão Preto Medical School, University of São Paulo and Department of Immunology and Microbiology at Faculdade de Medicina de Teresópolis between January 2010 and December 2020. Inclusion criteria for this study included a diagnosis of NF1 according to the NIH Consensus Development Conference diagnostic criteria. The medical history data collected through the review of medical records included age at diagnosis, sex, race/ethnicity, and clinical characteristics of NF1. Regarding PNs, we analyzed the localization, classification (asymptomatic, symptomatic operable, or symptomatic inoperable), malignancy, and others. We also described the main manifestations related to PNs, classified as physical disfigurement, orthopedic complaints, airway difficulties, gastrointestinal complaints, awkward gait, orthopedic, and others. This study was approved by the Institutional Review Boards of each center and performed in accordance with the 1964 Declaration of Helsinki. This project was funded by AstraZeneca.

## 3 STATISTICAL ANALYSES

The patient's baseline characteristics were digitized in an Excel spreadsheet, then imported into the SAS version 9.4 program to perform statistical analysis. The qualitative variables were summarized considering the absolute and relative frequencies, whereas the quantitative variables were summarized considering the measures of central position and dispersion. The variables are presented in table format.

## 4 RESULTS

**FIGURE 1:**
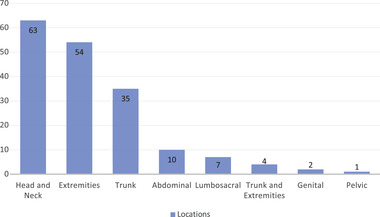
Number and predominant locations of plexiform neurofibromas in patients with neurofibromatosis type 1

This retrospective study included 491 patients (281 females and 210 males) with NF1. The median age at the time of the study was 30 years (range: 3 to 87 years), and 80.2% (305/380) were white. NIH criteria diagnosis main feature was CALMs, observed in 91% of patients (434/477). The patient's characteristics are summarized in Table 1. The prevalence of PNs in our cohort was 38.2% (176/460 patients). The median patient age with PNs was 30 years (range: 6 to 83 years). In terms of the distribution of PN throughout the body, 35.8% presented a PN located in the head and neck, 30.6% in the extremity, 19.9% in the trunk, and the remaining 13.7% in the abdomen, lumbosacral, genital, or pelvic region. Four patients had combined PN located in the trunk and extremity (Figure 1). PNs biopsy data were available in only 52 patients (29.5%), while magnetic resonance imaging (MRI) of PNs was performed in only 61 patients (34.6%).

**TABLE 1:** Characteristics of disease

Characteristics of NF1 patients	Values	%
Median age in years (range)	30 (3 to 87)	
Gender		
Male	210	42.77
Female	281	57.23
NIH criteria diagnosis		
CALMs	434/477	90.9
Axillary freckling	282/463	60.9
Inguinal freckling	126/419	30
Cutaneous neurofibromas	331/480	68.9
Plexiform neurofibroma	176/460	38.2
Lisch nodules	162/335	48.3
Optic glioma	55/394	13.9
Pseudoarthrosis	16/377	4.24
Sphenoid dysplasia	16/419	3.8
PN Location (N = 176)		
Head and neck	63	35.8
Extremity	54	30.68
Trunk	35	19.89
Abdomen	10	5.68
Lumbosacral	7	3.98
Genital	2	1.14
Pelvic	1	0.57
Trunk and extremity	4	2.27
Diagnostic test		
PN biopsy	52/176	29.5
PN MRI	61/176	34.6
PNs classification (N = 137)		
Asymptomatic	37	25.1
Symptomatic operable	33	22.4
Symptomatic inoperable	77	52.3
PNs related complication (N = 86)		
Disfigurement	41	47.6
Orthopedic	13	15.1
Awkward gait	11	12.7
Paresthesias	9	10.4
Speech problem	3	3.4
Medular compression	3	3.4
Weakness	2	2.3
Gastrointestinal	2	2.3
Airway difficulties	2	2.3

The PNs were classified as asymptomatic in 37 (25.1%), symptomatic operable in 33 (22.4%), and symptomatic inoperable in 77 patients (52.5%). Symptomatic inoperable tumors were found mostly in the head and neck (65%) and trunk (38%) while 65% of PNs located in extremities were classified as asymptomatic or symptomatic operable. Twenty‐five patients with symptomatic operable PN underwent surgery, a mean of 1.44 (1 to 4) surgeries per patient (SD, 0.87). Detailed information on surgical procedures was found in 20 patients. Complete excision was achieved in 13 of the 20 cases.

**FIGURE 2:**
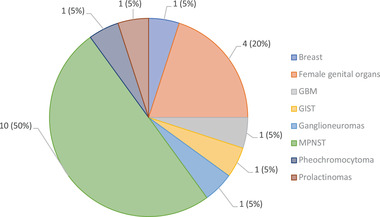
Types of cancer in patients with neurofibromatosis type 1

Data on PNs complications were available for 86 patients. The most common issue were disfigurement (41 patients), orthopedic (13), and awkward gait (11). A total of 43.3% (49/113) of patients reported pain associated with PN. Among 342 patients with NF1 analyzed, 20 developed neoplasms with a mean of 36.5 (range: 11 to 61) years (SD, 13.9). Of referred above, ten patients (50%) had MPNST, 4 had female genital cancer, and 6 developed other cancer types (Figure 2). Thus, the prevalence of MPNST in our study was 2.9%.

Considering patients under 20 years of age, we found a prevalence of PN of 29.8% (37/124). Of these, 48.3% (15/31) were considered symptomatic and inoperable, while 16.1% (5/31) were considered symptomatic and operable. Sixty percent of these patients (12/20) had pain associated with PNs. Regrettably, two patients died, one secondary to respiratory failure (cervical PN) at 7 years old and the development of MPNST at 13 years, respectively.

## 5 DISCUSSION

In the present study, we report the data from 491 patients diagnosed with NF1. As expected, CALMs, cutaneous neurofibromas, and freckling were the clinical aspects most observed in our NF1 population, found in 90.3, 68.9, and 60.9% respectively (Boulanger & Larbrisseau, 2005; Gross & Widemann, 2020). Once they are present in almost all patients with NF1 and are commonly the first visible sign of the disease, CALMs are considered the most important clinical criteria in patients with NF1 (Hernández‐Martín & Duat‐Rodríguez, 2016).

On the other hand, bearing in mind the impact on clinical manifestations, neurofibromas are considered one of the most important clinical manifestations in NF1. They are classified as benign neoplasms that originate from the peripheral nerve sheath, consisting of Schwann cells, perineural cells, fibroblasts, mast cells, axons, and secreted collagen (Korf, 1999; Rodrigues et al., 2014; Staser et al., 2012). Neurofibromas can be divided into cutaneous or subcutaneous and PNs. Although frequent in patients with NF1, PN is not a pathognomonic finding of this syndrome (Batista et al., 2015; Korf, 1999; Nguyen et al., 2011). PNs are thought to be congenital and occur in approximately 30% of children with NF1 and are recognized as benign tumors, highly vascularized, and locally invasive (Darrigo et al., 2007; Packer et al., 2002). Some lesions may be quiescent for an extended period, while others may grow aggressively, making these tumors an important cause of clinical complications in NF1, mainly in childhood and adolescence (Dombi et al., 2007; Geller et al., 2009).

In our study, 176 patients (38%) presented with PN. This is in accordance with previously reported studies (Boulanger & Larbrisseau, 2005; Korf, 1999). The most frequent location of the PNs was the head and neck (35.8%), followed by extremities (30.6%), trunk (19.9%), and abdomen (5.7%); (Figure 1). Regarding the classification of the PNs collected from 147 out of 491 patients, most of them (52.5%) were considered symptomatic and inoperable, while 22.4% were classified as symptomatic and operable. The management of these tumors included clinical follow‐up and surgical removal when there is involvement of a vital organ or suspicion of malignant transformation. Surgical removal outside these conditions is not indicated since tumors are rarely fully resected, as they are usually large and infiltrate adjacent tissues. Furthermore, residual lesions usually grow rapidly again, and there is no conventional medical approach to surgery in these patients (Korf, 1999; Pu & Vasconez, 2004; Wise et al., 2002). Thus, excision in the context of PNs is challenging and should be indicated with carefulness. Also, 48% of pediatrics and adolescents with PNs were classified as having symptomatic and inoperable tumors in our cohort. Sadder, 60% of these cases reported pain associated with PN. Several studies have demonstrated the impact between PNs in pediatric patients, and social–emotional problems (Martin et al., 2012; Wolters et al., 2015). Spontaneous tumor shrinkage has been reported in a previously study, however, no spontaneous reduction was reported in our cohort (Dombi et al., 2007).

Consequently, considering that PNs can be life‐threatening, new therapeutic proposals are highly desirable. The first approved drug for NF1‐related inoperable PN, selumetinib, a MEK inhibitor that has shown activity against several advanced cancers (Dombi et al., 2016; Mukhopadhyay et al., 2021). Gross *et al*. recently published a phase 2 study with 50 NF1 pediatric patients with symptomatic inoperable PNs. The patients had a median of three neurofibroma‐related symptoms; most of the disfigurement. A total of 68% of patients presented a confirmed partial response, and most of them had a durable response (lasting ≥ 1 year). Interestingly, responses were independent of age, tumor volume, and progression status at baseline, and the tumor's location (Gross et al., 2020; Killock, 2020). Recently, FDA approved selumetinib for the treatment of pediatric patients of 2 years of age and older with NF1, who have symptomatic, inoperable PNs (Casey et al., 2021).

Another promising drug, cabozantinib, a multiple tyrosine kinase receptor inhibitor, has had its data recently published (Fisher et al., 2021). In this study, the authors demonstrated that 42% of patients presented a partial response (defined as having greater than a 20% decrease in tumor volume), and 11 had stable disease after 12 cycles of treatment, indicating its potential to reduce the volume of the tumor as well as the associated pain (Fisher et al., 2021). Other studies with different drugs, such as mirdametinib, trametinib, and binimetinib, are ongoing with encouraging results (Mueller et al., 2020; Perreault et al., 2019; Weiss et al., 2021).

NF1 patients are at a significantly increased risk of developing certain types of cancer including MPNST, astrocytoma, pheochromocytoma, breast cancer, and juvenile myelomonocytic leukemia (Korf, 2000). Among these, MPNST, a highly aggressive sarcoma, is considered the main neoplastic lesion in patients with NF1 (Evans, 2002). MPNSTs classically emerge in pre‐existing PNs, however, some authors have demonstrated the development of MPNSTs in patients without previous PNs.

Patients with MPNSTs commonly present with pain and neurologic deficits and have a poor prognosis (Evans, 2002). In our study, MPNST was diagnosed in 10 (2.9%) patients, and all but two presented a previous PN. In contrast, some studies have reported an incidence of approximately 10% of MPNST in NF1 patients (Evans, 2002; Tucker et al., 2005). Despite the difference, our result is consistent with data published previously by our group (Darrigo et al., 2007). Some possible explanations for this disparity may be the lower availability of diagnostic imaging in our centers and a high rate of loss to follow‐up observed. Although we had only 10 cases in our group, three died, while another four had a follow‐up loss.

A subset of another 10 subjects had additional NF1‐related tumors (Figure 2), including neoplasms in female genital organs (n = 4), multiform glioblastoma (n = 1), breast cancer (n = 1), gastrointestinal stromal tumor (n = 1), pheochromocytoma (n = 1), ganglioneuroma, and prolactinoma (n = 1). In our series, optic nerve gliomas were observed in 13.9% (55/339) of patients, likewise observed in the literature (Listernick et al., 2007). Overall, six patients with NF1‐related tumors died at a median age of 28.5 years (range: 11 to 46).

Some limitations of this study were the absence of clinical assessment using a standardized phenotypic checklist and a high rate of loss to follow‐up, as mentioned before.

Finally, despite recent advances in diagnosis and treatment for patients with NF1, there are many barriers to access these high‐cost strategies in low‐ and middle‐income countries. For instance, regarding the healthcare approach in the NF1 setting, we have two distinct public policies in Brazil: (1) private, regulated by ANS (Supplementary National Health Agency) which provides middle‐ and high‐income population, and (2) public (Health Ministry) which provides for the low‐income population. In general, both are expected to provide healthcare access for the population in general (with a possible disadvantage regarding the latter). Promoting a greater NF1 awareness for both agencies, as well as improving financial public and private policies regarding disease care and research, would all be key approaches for NF1 relief.

In addition, considering NF1 as a rare disease, collaborative studies worldwide will be essential to better understand the clinical and epidemiological aspects of PNs, contributing to cutting‐edge strategies in the treatment of these patients.

## 6 CONCLUSIONS

Although there is an important number of patients affected by NF1 in Brazil and other low‐ and middle‐income countries, there is little data in the literature regarding the behaviors of the benign and malignant lesions of the syndrome. We present here the results of a retrospective chart review of 491 NF1 patients with a total of 176 PNs. In summary, our results are comparable to the results reported from higher‐income countries and international registries. Special attention, particularly in pediatric patients, should be given to symptomatic and inoperable PNs, since they have high morbidity and potentially deadly complications.

## CONFLICT OF INTEREST

L.G.D.J received research grants from AstraZeneca. The remaining authors declare no conflict of interest.

## FUNDING INFORMATION

This project was supported by the AstraZeneca company.

### PEER REVIEW

The peer review history for this article is available at https://publons.com/publon/10.1002/brb3.2599

## Data Availability

The data that support the findings of this study are available on request from the corresponding author. The data are not publicly available due to privacy or ethical restrictions.
